# Prevalence of Anemia among Older Adults Residing in the Coastal and Andes Mountains in Ecuador: Results of the SABE Survey

**DOI:** 10.1155/2017/4928786

**Published:** 2017-02-21

**Authors:** Carlos H. Orces

**Affiliations:** Laredo Medical Center, Department of Medicine, 1700 East Saunders, Laredo, TX 78041, USA

## Abstract

*Objectives*. To estimate the prevalence of anemia and its determinants among older adults in Ecuador.* Methods*. The present study was based on data from the National Survey of Health, Wellbeing, and Aging. Hemoglobin concentrations were adjusted by participants' smoking status and altitude of residence, and anemia was defined according to the World Health Organization criteria (<12 g/dL in women and <13 g/dL in men). Gender-specific logistic regression models were used to examine the association between demographic and health characteristics and anemia.* Results*. A total of 2,372 subjects with a mean age of 71.8 (SD 8.2) years had their hemoglobin measured, representing an estimated 1.1 million older adults. The crude prevalence of anemia was 20.0% in women and 25.2% in men. However, higher anemia prevalence rates were seen with advancing age among black women and subjects residing in the urban coast. Likewise, certain health conditions such as hypoalbuminemia, cancer in men, chronic kidney disease, iron deficiency, and low grade inflammation were associated with increased odds of having anemia.* Conclusions*. Anemia is a prevalent condition among older adults in Ecuador. Moreover, further research is needed to examine the association between anemia and adverse health-related outcomes among older Ecuadorians.

## 1. Introduction

Anemia is a common medical disorder in older adults associated with increased risk of functional impairment, hospitalization, and mortality [1, 2]. In general, the prevalence of anemia increases with advancing age and is higher among men and black populations [3]. Gaskell et al. reported that the mean prevalence of anemia among older adults in developed countries was 12% in community-dwelling older adults, 40% in hospital admissions, and 47% in nursing homes residents [4]. Previous studies have documented that the etiology of anemia in older adults appears to be multifactorial, with nutritional anemias, chronic kidney disease, and chronic inflammation found in two-thirds of the cases and unexplained anemia of aging in one-third of the cases [3, 5].

While the prevalence of anemia has been described in children and women of reproductive age in Ecuador, there is scarce epidemiological data about this condition in older adults [6]. In the 1980s, a study conducted by Cañizares et al. described that the crude prevalence of anemia was 21% among 2,652 subjects aged 2 to 75 years in Ecuador. Notably, the prevalence of anemia differed considerably according to areas of the country. In fact, 30.2% of subjects residing in rural areas were classified as having anemia as compared with 4.1% of those from urban areas of the country [7]. Moreover, life expectancy has gradually increased in Ecuador over the past decades. For instance, Ecuadorians were expected to live 64.6 years in 1980–1985 and will reach 78.3 years by 2025–2030 [8]. These demographic changes may increase considerably the number of individuals at risk for anemia in the country. Therefore, this study aimed to estimate the prevalence of anemia in a nationally representative sample of adults aged 60 years and older.

## 2. Material and Methods

The present study was based on data from participants in the first National Survey of Health, Wellbeing, and Aging (Encuesta Nacional de Salud, Bienestar, y Envejecimiento) conducted between 2009 and 2010. This survey is a probability sample of households with a least one person aged 60 years or older residing in the Andes Mountains and coastal regions of Ecuador. In the primary sampling stage, a total of 317 sectors from rural areas (<2,000 inhabitants) and 547 sectors from urban areas of the country were selected from the 2001 population Census cartography. In the secondary sampling stage, 18 households within each sector were randomly selected based on the assumption that at least one person aged 60 years or older lived in 24% and 23% of the households along the coast and Andes Mountains region, respectively. Between April and August 2010, a total of 2,375 participants underwent biochemical evaluation to determine their hemoglobin concentration. Survey methodology, including operation manuals, is publicly available [9].

### 2.1. Characteristics of Participants

Age and sex were self-reported. The race of participants was classified according to the following question. “Do you consider yourself to be White, Black, Mestizo, Mulatto, or Indigenous?” Body height in centimeters and weight in kilograms were measured and the body mass index was calculated (kg/m^2^). Subjects also reported their region (coast versus Andes Mountains) and area of residence (urban versus rural). Literacy was defined by answering affirmatively to the question “Can you write and read a message?” Smoking status was classified as current, former, and never. Self-reported health was grouped as excellent to good and fair to poor. The following activities of daily living (ADLs) were included in the present study: walking across a room, dressing, bathing, eating, getting in and out of bed, and using the toilet. Those participants who needed help or were unable to perform one or more of the ADLs as a result of health problems were considered functionally impaired. Cognitive status was evaluated by the abbreviated Mini Mental State Examination (MMSE). This modified MMSE was developed by Icaza and Albala to identify the MMSE questions that could best explain cognitive deterioration. The abbreviated MMSE was developed with nine variables instead of the 19 original MMSE variables. A cut-off point of 12 or less was defined to identify people with cognitive impairment [10]. Subjects were defined as having diabetes if they had been previously diagnosed by a physician with this condition or if fasting plasma glucose was ≥126 mg/dL [11]. Moreover, the following physician-diagnosed chronic conditions were self-reported: cancer, chronic obstructive pulmonary disease (COPD), heart disease, and arthritis.

### 2.2. Laboratory Data

Laboratory data collected in the SABE survey were analyzed at NetLab (Quito, Ecuador). Hemoglobin concentrations were measured with an automated blood cell counter. Serum ferritin and vitamin B_12_ levels were determined by using the heterogeneous immunoassay method. High sensitive C-reactive protein (CRP) levels were calculated with automated turbidimetry and serum albumin and creatinine levels were measured by using automated photometry.

### 2.3. Definition of Anemia

Hemoglobin concentrations were adjusted by participants' smoking status and altitude of residence as recommended by the World Health Organization (WHO). Subsequently, anemia was defined by the WHO criteria as a hemoglobin concentration < 13 g/dL in men and <12 g/dL in women [12]. The mean red blood cell corpuscular volume (MCV) was classified as microcytic, normocytic, and macrocytic according to MCV levels < 80 fl, 80–100 fl, and >100 fl, respectively [13].

Ferritin, a marker of iron storage may be spuriously elevated in inflammatory conditions. Therefore a ferritin cut-off of 45 ng/mL has a higher sensitivity for iron deficiency anemia in older adults [14]. On the contrary, a serum ferritin level > 200 ng/mL serves to confirm that anemia is not related to iron deficiency [15, 16]. Consequently, older adults with these ferritin cut-off levels were classified as having iron deficiency anemia and anemia of chronic disease, respectively. Creatinine clearance (CrCl) was calculated according to the Cockroft-Gault equation and subjects with a CrCl < 30 mL/min were classified as having anemia of chronic kidney disease [3, 17]. In the present analysis, a CRP level ≥ 5 mg/L was the cut-off used to define low grade inflammation [18]. Vitamin B_12_ deficiency was defined as serum B_12_ levels < 200 pg/mL and hypoalbuminemia as a serum albumin concentration < 3.5 g/dL [3, 19].

### 2.4. Statistical Analysis

Chi-square for categorical variables and *t*-test for continuous variables were used to compare the characteristics of the participants by gender. The crude prevalence of anemia stratified by sociodemographic and health characteristics of participants and the MCV distribution according to selected causes of anemia were presented as percentages. Moreover, gender-specific logistic regression models adjusted for age, race, residence, literacy, and smoking status were created to examine the associations between demographic characteristics and health status of the participants and anemia. Results of the multivariate model are presented as odds ratios (OR) with their 95% confidence intervals (95% CI). All analyses used sample weights to account for the complex survey design and to report anemia prevalence nationwide. Statistical analyses were performed using SPSS, version 17 software (SPSS Inc., Chicago, IL).

## 3. Results

**Figure 1:**
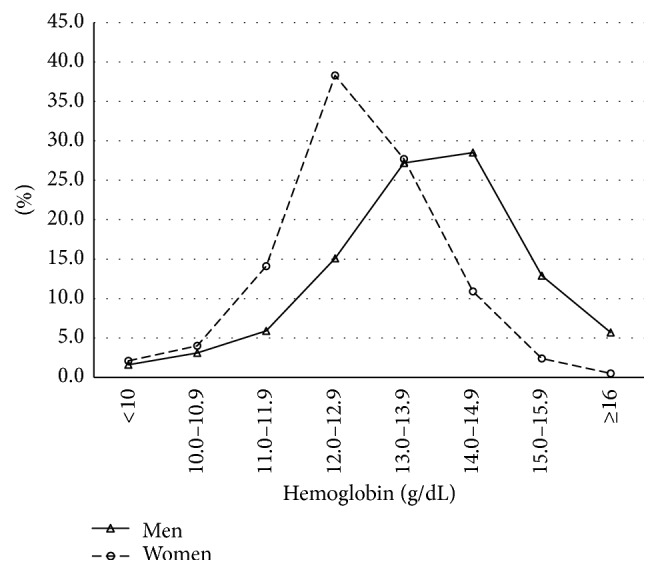
Hemoglobin concentrations among older adults in Ecuador, SABE II survey.

A total of 2,372 subjects with a mean age of 71.8 (SD 8.2) years had their hemoglobin measured, representing an estimated 1.1 million older adults residing in the coastal and Andes Mountains regions in Ecuador. As shown in Table 1, the age, area of residency, and race of participants were similarly distributed by gender. In general, higher proportions of men were smokers, literate, and reported good to excellent health. In contrast, women had higher rates of obesity, ADL's limitations, cognitive impairment, and comorbidities. Laboratory data revealed no significant gender differences regarding CRP, albumin, and ferritin concentrations. However, vitamin B_12_ levels were considerably lower among men. As shown in Figure 1, women tended to have lower hemoglobin concentrations than those in men. For instance, 58.5% of women had hemoglobin concentrations less than 13 g/dL as compared with 25.2% in men. However, only 2.1% and 1.6% of women and men had hemoglobin levels less than 10 g/dL, respectively.

Overall, 20.0% of women aged 60 years and older were defined as having anemia in Ecuador. Nevertheless, the prevalence of anemia among women was particularly increased in blacks and residents from the urban coast. Moreover, subjects with certain health conditions such as being underweight, chronic kidney disease, hypoalbuminemia, and iron deficiency had significant higher anemia prevalence rates. After adjustment for potential confounders, hypoalbuminemia, chronic kidney disease, being underweight, and iron deficiency were variables independently associated with 5.1-, 4.4-, 2.4-, and 2.1-fold increased odds of having anemia among older women, respectively (Table 2).

Anemia was prevalent in 25.2% of older men in Ecuador. Moreover, the prevalence of anemia in men increased with advancing age and was higher among those residing in the urban coast as compared with residents from other areas of the country. Likewise, subjects with hypoalbuminemia, those diagnosed with cancer, and participants defined as having chronic kidney disease, iron deficiency, and low grade inflammation had higher prevalence of anemia than those who did not. Notably, after adjustment for covariates, older men with hypoalbuminemia and those diagnosed with cancer were 6.8 and 6.5 times more likely to have anemia as compared with those without hypoalbuminemia, respectively (Table 3).

**Figure 2:**
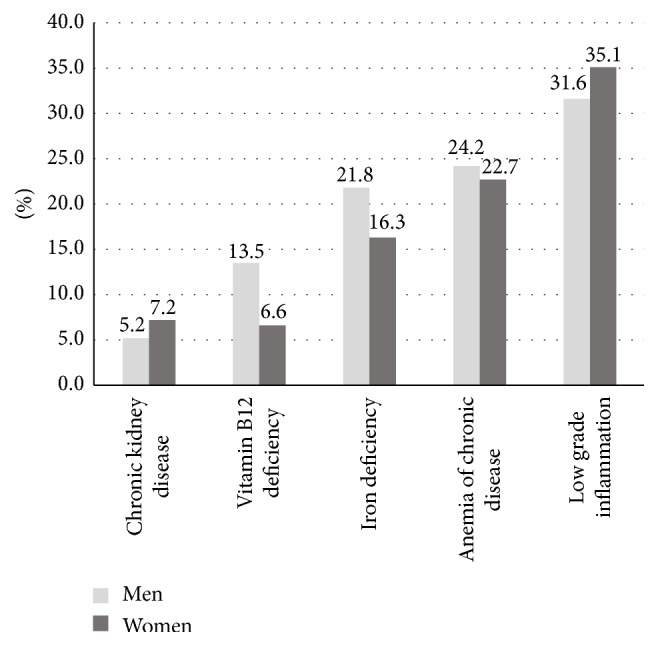
Selected causes of anemia among older adults in Ecuador, SABE survey.

Figure 2 shows the distribution of nutritional and nonnutritional causes of anemia in older Ecuadorians. Among nutritional anemias, iron deficiency was the leading cause of anemia in 21.8% of men and 16.3% of women, whereas 13.5% of men and 6.6% of women had low vitamin B_12_ concentrations. Although chronic kidney disease was associated with increased odds of having anemia in both genders, only 5.2% of men and 7.2% of women were classified as having anemia of chronic kidney disease. Of interest, 24.2% of men and 22.7% of women had ferritin levels > 200 ng/mL, which is highly suggestive of anemia of chronic disease. Of relevance, one-third of older adults with anemia had evidence of low grade inflammation.  Table 4 shows the MCV distribution among older adults with anemia in Ecuador. In general, 88% of subjects with anemia had a normal MCV index. In general, normocytic anemia was predominantly seen in participants with kidney disease, chronic disease, and low grade inflammation. Moreover, 13.9% of men and 14.7% of women with iron deficiency anemia had evidence of microcytosis. On the contrary, a macrocytic morphology was present in only 3.1% of subjects with vitamin B_12_ deficiency.

## 4. Discussion

The present results indicate that anemia represents a moderate public health problem among older adults in Ecuador according to the WHO criteria (prevalence from 20.0% to 39.9%) [12]. Overall, one in five subjects aged 60 years and older was defined as having anemia in Ecuador. Moreover, increased anemia prevalence rates were predominantly seen with advancing age, in men, black women, and subjects residing in the urban coast. Interestingly, the geographic distribution of anemia found in the present study contrast with those results previously reported by Cañizares et al. in the 1980s in which a significant higher prevalence of anemia was described in the rural areas of the country [7]. It may be postulated that the increased prevalence of anemia among older adults in urban areas of the country may be explained by changes in the demographic distribution of the population over time. For instance, the percentage of adults aged 65 years and older who lived in the urban areas of the country increased from 4.3% in 1980 to 5.7% in 2010. Similarly, the aging index in the urban areas increased from 14.5 to 28.2 per 100 persons during the same period of time [8].

Notably, up to 42.0% of older black women were found to be anemic in Ecuador. This finding may be associated with an increased prevalence of hemoglobin variants in Afro-Ecuadorians. Indeed, a recent study reported that 22.0% of Afro-Ecuadorians from the province of Imbabura had a hemoglobin variant. Of these, hemoglobin S was the most frequently reported variant representing 14.0% of the cases. Moreover, it appears that the prevalence of hemoglobinopathies in Ecuador is higher than that reported in other Latin America countries [20]. Consistent with the present findings, prior studies have demonstrated lower hemoglobin levels among blacks of all ages even after accounting for comorbidities, behavioral risk factors, nutritional intake, and iron status [3, 21].

In Ecuador, older adults had higher anemia prevalence rates as compared with those previously described among older adults in the United States (10.6%), the Chianti area in Italy (11.3%), and Mexico (13.9%) [3, 22, 23]. Likewise, a recent population-based study conducted among older adults in Latin America reported lower anemia prevalence rates in Cuba (19.2%) and Venezuela (9.8%). On the contrary, up to 37.3% and 32.1% of older adults in Dominican Republic and Puerto Rico were classified as having anemia, respectively [24]. The present results indicate that the prevalence of anemia in older Ecuadorians is comparable to that recently described among subjects aged 65 years and older in Portugal (21.0%) [25].

Of relevance, certain health conditions such as hypoalbuminemia, chronic kidney disease, iron deficiency, and cancer in men were associated with increased anemia prevalence rates. In agreement with these findings, Salive et al. reported that, among participants in the Established populations for the Epidemiologic Studies of the Elderly, men and women with hypoalbuminemia were 6.1 and 5.2 as likely to have anemia, respectively [19]. The strong relationship between hypoalbuminemia and anemia has been mediated through the prevalence of chronic disease and nutritional factors [19]. Similarly, chronic kidney disease among older Ecuadorians was also associated with a markedly increased prevalence of anemia in both genders. Bowling et al. using data from the National Health and Nutrition Examination Survey recently described that subjects aged 60–69, 70–79, and 80 years and older with an estimated glomerular filtration rate (eGFR) of <45 mL/min were 3.8, 3.2, and 2 times as likely to have anemia as compared with their counterparts with an eGFR ≥ 60 mL/min, respectively [26]. Despite the strong relationship between decreased eGFR and anemia, chronic kidney disease accounted for a small proportion of older adults with anemia in Ecuador, which is consistent with results from a prior investigation [3].

In Ecuador, one-third of older adults with anemia had evidence of iron and vitamin B_12_ deficiency. Overall, the prevalence of iron deficiency anemia among older Ecuadorians was higher than that reported among older adults in the US and Mexico [3, 23]. This marked difference in iron deficiency anemia prevalence rates may be partly explained by study methodology differences as a higher ferritin cut-off level was used as an indicator of iron deficiency anemia in the present study [14]. While some cases of iron deficiency results from diet, the etiology of iron deficiency anemia in older adults is most likely associated with gastrointestinal lesions. Rockey and Cello demonstrated that, among subjects evaluated for idiopathic iron deficiency anemia, gastrointestinal endoscopy revealed a least one lesion potentially responsible for blood loss in 62% of cases. The most frequent abnormality in the upper gastrointestinal tract was peptic ulcer and cancer was the most common colonic lesion [27]. In Ecuador, it may be postulated that the increased prevalence of anemia found among older men diagnosed with cancer may be associated with iron, vitamin B_12_ deficiency, and chronic inflammation. Indeed, previous research has shown that both atrophic gastritis and* H*.* pylori* infection may result in vitamin B_12_ and iron deficiency [28, 29]. Similarly, these gastric pathologies increase considerably the risk of gastric cancer [30, 31]. Despite this evidence, further research is needed to confirm these associations. Moreover, the increased prevalence of vitamin B_12_ deficiency among older men in Ecuador is consistent with results from previous investigations reporting higher vitamin B_12_ deficiency prevalence rates among older men in the US and Chile [32, 33]. In Ecuador, the reasons for the gender difference in vitamin B_12_ concentrations are unknown. However, vitamin B_12_ dietary sources and supplements, which were not examined in the present study, may partly explain this finding. Of clinical relevance, only 3% of older Ecuadorians with vitamin B_12_ deficiency had evidence of macrocytic anemia. Likewise, Loikas et al. demonstrated in a population-based study among older from Finland that the presence of macrocytosis or macrocytic anemia did not increase the odds of having vitamin B_12_ deficiency, which is consistent with the present results [34].

Interestingly, one-third of older Ecuadorians with anemia had evidence of low grade inflammation. Nonnutritional anemia in older adults has been documented to result from an interaction between an increased inflammatory milieu and age-related comorbidities. Among the inflammatory markers, IL-6 leads to iron-limited hematopoiesis with increased hepcidin levels and poor iron incorporation into the developing erythrocytes. In addition, tumor necrosis factor *α*, in a different pathway, leads to erythropoietin resistance of the hematopoietic stem cells, increasing its demand and eventually exceeding the ability of the kidney to maintain adequate levels to sustain normal hemoglobin [15].

The present study has several limitations that should be mentioned. First, because of the cross-sectional study design, the association of anemia with certain demographic and health characteristics does not necessarily indicate causation. Second, the evaluation of nutritional and nonnutritional anemia should be considered preliminary. For instance, iron and vitamin B_12_ anemia were defined exclusively according to ferritin and vitamin B_12_ levels available in the SABE survey, respectively. Moreover, other potential causes of anemia affecting older adults such as folate deficiency and myelodysplastic syndromes requiring cytogenetic and bone marrow analyses were not evaluated in the present study [34]. Third, most of the participant's comorbidities and lifestyle variables were self-reported, which may be a source of recall bias. Fourth, the present findings may be only generalized to older adults residing in the coastal and Andes Mountains regions of the country. Despite these limitations, the present study reports national estimates of anemia among older adults.

In conclusion, anemia is a prevalent condition among older adults in Ecuador. Moreover, further research is needed to examine the association between anemia and adverse health-related outcomes among older Ecuadorians.

## Figures and Tables

**Table 1:** Demographic and health characteristics of participants in the SABE survey.

	Men (*n* = 1,065)	Women (*n* = 1,307)	*P* value
Age groups (years), %			.480
60–69	47.5	46.2	
70–79	31.6	34.5	
≥80	20.9	19.3	
Race, %			.881
Indigenous	10.0	11.1	
Black	3.8	3.4	
Mestizo	70.6	69.1	
Mulatto	3.8	3.3	
White	11.8	13.1	
Area of residency, %			.149
Urban Andes Mountains	28.3	30.8	
Urban coast	36.1	37.5	
Rural Andes Mountains	20.2	20.3	
Rural coast	15.4	11.4	
Literacy, %	76.7	64.7	<.0001
Smoking, %			<.0001
Current	20.5	3.3	
Former	49.1	12.4	
Never	30.4	84.3	
BMI (kg/m^2^), %			<.0001
Underweight	2.3	2.0	
Normal	49.7	32.9	
Overweight	37.8	38.5	
Obesity	10.2	26.6	
Self-reported health, %			<.0001
Excellent to good	30.1	20.2	
Fair to poor	69.9	79.8	
ADL's limitations, %	22.9	29.5	<.005
Cognitive impairment, %	16.6	25.9	<.0001
Comorbidities, %			
Diabetes	13.2	19.6	<.005
Cancer	2.2	3.5	.117
COPD	6.5	9.7	.032
Heart disease	12.2	15.8	.086
Arthritis	20.6	43.0	<.0001
Number of comorbidities, %			<.0001
0	58.2	37.3	
1	31.5	39.9	
≥2	10.3	22.8	
CRP, mg/L (SD)	4.9 (8.9)	5.1 (9.0)	.652
Creatinine clearance mL/min (SD)	66.2 (20.5)	66.6 (23.4)	.629
Albumin, g/dL (SD)	4.3 (0.3)	4.3 (0.3)	.950
Vitamin B_12_, ng/mL (SD)	423.9 (234.6)	527.7 (261.6)	<.0001
Ferritin, ng/mL (SD)	172.5 (164.5)	165.9 (159.0)	.325

**Table 2:** Prevalence of anemia among older women in Ecuador.

Characteristics	Crude prevalence	OR (95% CI)^*∗*^
Age groups (years), %		
60–69	17.8 (14.0, 22.2)	1.00
70–79	20.5 (16.5, 25.3)	1.17 (1.15, 1.19)
≥80	24.1 (18.7, 30.5)	1.37 (1.35, 1.40)
Race, %		
Indigenous	19.0 (11.6, 29.7)	0.69 (0.68, 0.71)
Black	42.4 (24.4, 62.7)	2.19 (2.12, 2.27)
Mestizo	17.8 (14.9, 21.1)	0.66 (0.64, 0.67)
Mulatto	24.5 (11.5, 44.6)	0.91 (0.87, 0.94)
White	25.3 (18.3, 33.8)	1.00
Area of residency, %		
Urban Andes Mountains	18.4 (14.3, 23.4)	1.58 (1.54, 1.62)
Urban coast	22.5 (18.3, 27.3)	1.94 (1.89, 1.99)
Rural Andes Mountains	20.8 (15.0, 28.1)	1.67 (1.63, 1.72)
Rural coast	14.2 (9.1, 21.6)	1.00
Literacy, %		
Yes	19.5 (16.6, 22.7)	1.00
No	20.8 (16.2, 26.2)	1.09 (1.08, 1.11)
Smoking, %		
Current	32.8 (16.7, 54.2)	1.80 (1.75, 1.86)
Former	19.6 (13.4, 27.8)	0.99 (0.97, 1.01)
Never	19.5 (16.7, 22.5)	1.00
BMI (kg/m^2^), %		
Underweight	34.5 (19.1, 54.1)	2.41 (2.31, 2.51)
Normal	28.5 (23.0, 34.6)	2.14 (2.10, 2.18)
Overweight	14.9 (11.5, 19.0)	0.96 (0.94, 0.98)
Obesity	16.1 (12.2, 21.0)	1.00
Self-reported health, %		
Excellent to good	13.1 (9.1, 18.4)	1.00
Fair to poor	21.8 (18.8, 25.1)	1.97 (1.93, 2.00)
ADL's limitations, %		
Yes	24.0 (19.2, 29.6)	1.43 (1.41, 1.45)
No	18.3 (15.4, 21.5)	1.00
Cognitive impairment, %		
Yes	24.3 (18.1, 31.7)	1.44 (1.42, 1.47)
No	18.4 (15.6, 21.5)	1.00
Diabetes		
Yes	22.6 (16.9, 29.4)	1.25 (1.24, 1.27)
No	19.3 (16.5, 22.4)	1.00
Cancer, %		
Yes	21.0 (10.7, 37.0)	1.15 (1.13, 1.19)
No	20.0 (18.1, 23.7)	1.00
COPD, %		
Yes	13.0 (6.0, 26.1)	0.54 (0.52, 0.55)
No	20.7 (18.1, 23.7)	1.00
Heart disease, %		
Yes	22.9 (15.5, 32.5)	1.27 (1.25, 1.29)
No	19.4 (16.8, 22.2)	1.00
Arthritis, %		
Yes	22.3 (18.1, 27.2)	1.52 (1.50, 1.54)
No	18.4 (15.3, 21.8)	1.00
CRP (mg/L), %		
<5	17.5 (14.7, 20.7)	1.00
≥5	26.8 (21.4, 33.0)	1.69 (1.67, 1.72)
Creatinine clearance, %		
≥30 mL/min	19.0 (16.4, 21.8)	1.00
<30 mL/min	57.2 (36.7, 75.5)	4.44 (4.30, 4.59)
Albumin (g/dL), %		
≥3.5	19.0 (16.5, 21.8)	1.00
<3.5	56.0 (23.2, 84.3)	5.01 (4.85, 5.18)
Vitamin B_12_ (ng/mL), %		
≥200	20.1 (17.4, 23.0)	1.00
<200	18.4 (11.4, 28.2)	0.87 (0.85, 0.89)
Ferritin (ng/mL), %		
<45	34.5 (25.1, 45.2)	2.17 (2.13, 2.21)
≥45	18.4 (15.9, 21.3)	1.00
Ferritin (ng/mL), %		
≤200	20.4 (17.6, 23.6)	1.00
>200	18.5 (13.5, 24.8)	0.84 (0.83, 0.86)

^*∗*^Adjusted for age, race, area of residency, literacy, and smoking status.

**Table 3:** Prevalence of anemia among older men in Ecuador.

Characteristics	Crude prevalence	OR (95% CI)^*∗*^
Age groups (years), %		
60–69	19.5 (15.1, 24.9)	1.00
70–79	27.9 (22.4, 34.1)	1.56 (1.54, 1.59)
≥80	34.2 (26.8, 42.6)	1.81 (1.78, 1.84)
Race, %		
Indigenous	15.6 (8.7, 26.4)	0.63 (0.61, 0.65)
Black	26.3 (14.1, 43.9)	1.01 (0.97, 1.05)
Mestizo	26.7 (22.6, 31.3)	1.16 (1.14, 1.19)
Mulatto	19.5 (9.7, 35.1)	0.65 (0.62, 0.68)
White	24.9 (16.0, 36.7)	1.00
Area of residency, %		
Urban Andes Mountains	23.3 (17.8, 29.9)	0.94 (0.92, 0.96)
Urban coast	30.4 (24.3, 37.2)	1.42 (1.40, 1.45)
Rural Andes Mountains	19.3 (14.5, 25.3)	0.86 (0.84, 0.88)
Rural coast	24.5 (17.4, 33.5)	1.00
Literacy, %		
Yes	23.6 (20.0, 27.7)	1.00
No	30.7 (23.9, 38.7)	1.34 (1.32, 1.36)
Smoking, %		
Current	24.8 (17.5, 33.8)	1.12 (1.10, 1.14)
Former	26.2 (21.5, 31.6)	1.10 (1.08, 1.11)
Never	23.6 (18.8, 29.2)	1.00
BMI (kg/m^2^), %		
Underweight	35.0 (18.5, 56.1)	1.55 (1.48, 1.63)
Normal	29.5 (24.4, 35.1)	1.49 (1.45, 1.52)
Overweight	18.7 (14.3, 24.2)	0.82 (0.80, 0.84)
Obesity	19.0 (11.8, 29.3)	1.00
Self-reported health, %		
Excellent to good	18.2 (13.6, 23.9)	1.00
Fair to poor	28.2 (24.1, 32.7)	1.65 (1.62, 1.67)
ADL's limitations, %		
Yes	36.1 (28.8, 44.2)	1.71 (1.69, 1.74)
No	22.0 (18.5, 26.0)	1.00
Cognitive impairment, %		
Yes	34.6 (26.0, 44.5)	1.76 (1.72, 1.79)
No	21.9 (18.4, 25.8)	1.00
Diabetes		
Yes	25.6 (17.7, 35.6)	1.04 (1.02, 1.06)
No	25.2 (21.7, 29.0)	1.00
Cancer, %		
Yes	68.0 (42.8, 85.7)	6.53 (6.27, 6.80)
No	24.2 (21.0, 27.8)	1.00
COPD, %		
Yes	28.6 (18.4, 41.5)	1.07 (1.04, 1.10)
No	24.9 (21.5, 28.6)	1.00
Heart disease, %		
Yes	21.9 (14.6, 31.7)	0.65 (0.64, 0.67)
No	25.7 (22.2, 29.6)	1.00
Arthritis,%		
Yes	29.8 (22.5, 38.3)	1.29 (1.27, 1.31)
No	24.0 (20.5, 28.0)	1.00
CRP (mg/L), %		
<5	21.9 (18.3, 26.0)	1.00
≥5	37.5 (30.6, 45.0)	2.10 (1.24, 1.27)
Creatinine clearance (mL/min), %		
>30	23.8 (20.5, 27.4)	1.00
≤30	57.3 (37.8, 74.8)	2.98 (2.86, 3.11)
Albumin (g/dL), %		
≥3.5	24.4 (21.1, 28.0)	1.00
<3.5	71.1 (39.7, 90.2)	6.83 (6.52, 7.16)
Vitamin B_12_ (ng/mL), %		
≥200	25.7 (22.1, 29.6)	1.00
<200	22.7 (16.3, 30.8)	0.81 (0.80, 0.83)
Ferritin (ng/mL),%		
<45	43.9 (32.5, 56.1)	2.78 (2.73, 2.83)
≥45	22.6 (19.3, 26.1)	1.00
Ferritin (ng/mL), %		
≤200	26.7 (22.9, 31.0)	1.00
>200	21.5 (15.9, 28.5)	0.80 (0.79, 0.81)

^*∗*^Adjusted for age, race, area of residency, literacy, and smoking status.

**Table 4:** MCV distribution among older adults with selected causes of anemia in Ecuador.

	MCV < 80 fl	MCV 80–100 fl	MCV > 100 fl

Men			
Total	7.6 (2.3)	87.4 (3.0)	5.0 (2.2)
Chronic kidney disease	—	100.0 (0)	—
Vitamin B_12_ deficiency	1.7 (1.1)	94.3 (2.1)	4.0 (1.9)
Iron deficiency	13.9 (11.4)	86.1 (4.3)	—
Chronic disease	0.5 (0.4)	96.3 (1.4)	3.2 (1.3)
Low grade inflammation	1.8 (0.9)	96.8 (1.2)	1.4 (0.7)

Women			
Total	10.2 (2.4)	88.4 (2.5)	1.3 (0.7)
Chronic kidney disease	—	93.4 (4.6)	6.6 (4.6)
Vitamin B_12_ deficiency	2.6 (1.6)	95.3 (2.0)	2.2 (1.3)
Iron deficiency	14.7 (3.8)	85.3 (3.8)	—
Chronic disease	0.7 (0.5)	98.4 (0.7)	0.9 (0.5)
Low grade inflammation	5.8 (1.8)	93.4 (1.8)	0.9 (0.5)
